# Comparative analysis of the interactions of different *Streptococcus suis* strains with monocytes, granulocytes and the complement system in porcine blood

**DOI:** 10.1186/s13567-024-01268-z

**Published:** 2024-02-05

**Authors:** Haodan Zhu, Uwe Müller, Christoph Georg Baums, Sophie Öhlmann

**Affiliations:** 1 Institute of Bacteriology and Mycology, Centre for Infectious Diseases, Faculty of Veterinary Medicine, University of Leipzig, Leipzig, Germany; 2 Institute of Veterinary Medicine, Jiangsu Academy of Agricultural Sciences, Nanjing, China; 3 Institute of Immunology, Centre for Infectious Diseases, Faculty of Veterinary Medicine, University of Leipzig, Leipzig, Germany

**Keywords:** Opsonophagocytosis, autoaggregation, bacteremia, bactericidal assay, C3 deposition, oxidative burst, porcine monocyte

## Abstract

*Streptococcus suis* (*S. suis*) is an important porcine pathogen causing meningitis, arthritis, and septicemia. Serotypes 2 and 14 are the most common zoonotic ones worldwide, whereas serotypes 2, 9, and 7 are very important in pigs in Europe. To cause invasive infections *S. suis* needs to enter the bloodstream. Consequently, the immune response in blood represents an important line of defense and bacteremia plays a key role in the pathogenesis of invasive *S. suis* infections. We investigated the working hypothesis that *S. suis* strains of the same serotype but different clonal complex (CC) might exhibit substantial differences in the interaction with components of the immune system in porcine blood. The experimental design of this study includes comparative analysis of 8 virulent strains belonging to 4 serotypes with strains of the same serotype being genetically not closely related. Significant differences between two strains of the same serotype but different clonal complex were recorded in the flow cytometric analysis of association with different leukocytes for serotype 9 and 14. Our results demonstrate that the serotype 9 strain of CC94 shows significantly increased association with monocytes and survival in porcine blood of conventional piglets as well as a tendency towards decreased composition of C3 in plasma of these piglets in comparison to the serotype 9 strain of CC16. Correlation analysis of C3 deposition on the bacterial surface and survival in respective blood samples of 8-week-old piglets demonstrated a negative correlation indicating that C3 deposition is a crucial step to limit bacterial survival and proliferation of different *S. suis* pathotypes in the blood of these piglets. In summary, our results indicate that the capsule composition of a *S. suis* strain is not alone sufficient to determine association with leukocytes, activation of complement, induction of proinflammatory cytokines, oxidative burst, and bacterial survival in porcine blood. In this study, substantial differences in these host–pathogen interactions were observed between strains of the same serotype. Therefore, a more comprehensive characterization of the field isolates, including at least MLST analysis to determine the sequence type/clonal complex, is recommended.

**Supplementary Information:**

The online version contains supplementary material available at 10.1186/s13567-024-01268-z.

## Introduction

Invasive *S. suis* strains express a polysaccharide capsule as protection against phagocytosis [1, 2]. The composition and structure of the capsule determines the serotype (*cps*). Up to now 29 different *S. suis* serotypes have been described, of which *cps*2 is the most prevalent in association with disease in pigs and humans worldwide [3]. In Europe, *cps*9 has become the most important serotype causing main herd problems and substantial economic losses [4, 5]. A further important *cps* in some European countries is 7 [6–8]. In Germany, *cps*7 is emerging during the last years [6]. It is not only associated with porcine disease but was also recently found in blood cultures of human patients with septicemia and pneumonia in China [9]. In addition to serotyping numerous laboratories conduct multilocus sequence typing (MLST) to differentiate *S. suis* isolates [10, 11]. MLST analysis has demonstrated that strains of the same *cps* might belong to different clonal complexes (CC) and vice versa which is in agreement with horizontal transfer of the genes encoding biosynthesis of the capsule.

Meningitis, arthritis, endocarditis, and sudden death are important manifestations of *S. suis* infection [12]. To cause these pathologies, *S. suis* must enter the bloodstream and strive within this compartment. Different mechanisms are discussed how bacteremia might lead to meningitis. One is based on the modified Trojan horse theory, suggesting *S. suis* breaches the blood–brain or the blood-cerebrospinal fluid barrier bound to the surface of monocytes [13].

Different in vitro and in vivo experiments have shown that the complement system is an important host defense mechanism against *S. suis cps*2 [14–18]*.* As an example, the addition of the C3 convertase inhibitor vaccine virus complement control protein to *S. suis* infected porcine blood leads to a significantly increased proliferation of *S. suis* [18]. The complement system is crucial for control of *S. suis* bacteremia when no opsonizing IgG but high specific IgM levels are present [18]. Although *S. suis* expresses proteins involved in protection against reactive oxygen species (ROS) such as superoxide dismutase (SOD) [19] and Dps-like peroxide resistance (Dpr) [20, 21], our previous studies have indicated that generation of ROS by activated blood granulocytes plays an important role in host defense against *S. suis* [18].

The interaction of *S. suis* with the immune system has been mainly studied with *cps*2 strains of CC1. We wondered whether strains belonging to different serotypes and CCs show a comparable phenotype in their interaction with leukocytes, more precisely the association with monocytes and the induction of ROS and cytokines. We also compared their ability to activate the complement system and the survival in porcine blood after in vitro infection. For this we used 8 strains in various in vitro assays designed to address different aspects of bacteremia.

## Materials and methods

### Bacterial strains and growth conditions

*S. suis cps*2 strain 10 is an *mrp*^+^ *epf* ^+^ *sly* ^+^ strain of sequence type 1 that has been used by different groups successfully to induce disease experimentally [1, 22, 23]. Strain 10 and its capsule mutant strain 10*cps*ΔEF were kindly provided by Hilde Smith, DLO-Lelystad [1]. The second *cps*2 strain 483 is an *mrp*^+^ *epf*^−^ *sly*^−^strain of sequence type 28 that was isolated from the lung of a pig in Germany that had suddenly died due to *S. suis* infection [24]. Of note, this genotype was also isolated from the spleen of a further pig in this herd showing sudden death and *S. suis* was detected in pure culture and high content in both cases in different inner organs. Serotype 14 strains V3117/2 and TW078/11 were isolated from the brain of a pig with meningitis in Germany and from inner organs of a clinically diseased pig in the United Kingdom, respectively [25]. Serotype 9 strains 16085/3b and 8067 are highly virulent strains of sequence types 94 and 136, respectively [5, 26]. 16085/3b was isolated from the spleen of a pig with septicemia [26] while 8067 proved virulent in experimental infection of pigs [5] and was originally isolated from “CSF or blood culture” of a pig based on information provided in the NCBI nucleotide database (NCBI Reference Sequence: NZ_CZEL01000011.1). Serotype 7 strains 13–00283-02 (mrp^****^epf^−^*sly*^−^) of sequence type 29 and S5552/1 (*mrp*^−^*epf*^−^*sly*^+^) of sequence type 89 were isolated from the brains of pigs with meningitis in 2013 and 2010 in Germany, respectively [6] (Table 1). Bacteria were grown either on Columbia agar plates with 6% sheep blood (Oxoid, Wesel, Germany) or in Bacto^™^ Todd Hewitt Broth (THB) at 37 ℃ overnight, if not stated otherwise. *S. suis* glycerol stocks were prepared at the exponential growth phase (OD600 = 0.5) and stored at −80 ℃ in 15% glycerol as single-use aliquots.

**Table 1:** **Genotypic features as well as clinical background of** ***S. suis* strains investigated in this study**

*S. suis* strains	Serotype	Sequence type (MLST)	Clonal complex (MLST)	Clinical background	Years of isolation	Countries of isolation	Profile of virulence-associated genes	Virulence in experimental infection	References
10	2	1	1	tonsil	1982	Netherlands	mrp + /epf + /sly +	virulent	[1, 22, 23]
10cpsΔEF	2	1	1	capsule mutant	1999	Netherlands	mrp + /epf + /sly +	avirulent	[1]
483	2	28	27	toxic shock	2019	Germany	mrp + /epf-/sly-	not investigated	[24]
V3117/2	14	1	1	brain	2013	Germany	mrp + /epf + /sly +	virulent	[25, 34]
TW078/11	14	1552	1	invasive	2011	United Kingdom	mrp^s^/epf + /sly +	not investigated	[25]
16085/3b	9	94	94	septicemia	2016	Germany	mrp + /epf-/sly +	virulent	[26]
8067	9	136	16	meningitis	1996	Netherlands	mrp-/epf-/sly +	virulent	[5]
13-00283-02	7	29	29	brain	2013	Germany	mrp^****^/epf-/sly-	virulent	[6]
S5552/1	7	89		brain	2010	Germany	mrp-/epf-/sly +	not investigated	[6]

*mrp* variant: 747 bp for *mrp*^*S*^, 2400 bp for mrp^****^[6].

### Microbial adhesion to hydrocarbons (MATH) assay

Hydrophobicity of *S. suis* was evaluated by measuring bacterial adhesion to hexadecane (Sigma, H6703) following a previously described protocol with slight modifications [27, 28]. Briefly *S. suis* strains were cultured overnight and harvested by centrifugation (3900 × *g*, 10 min, 4 ℃). Pellets were resuspended in PBS and washed twice before adjusting the suspensions to an OD_600_ of 1 (OD_A_). Then, 2 mL of bacterial suspension was mixed with 400 µL of hexadecane and tubes were vortexed for 30 s. The mixture was allowed to separate into two phases for 30 min at room temperature. The aqueous phase was collected and OD_600_ (OD_B_) was measured. Cell surface hydrophobicity was calculated as follows: % hydrophobicity = [1—(OD_B_/OD_A_)] × 100.

### Autoaggregation assay

*S. suis* isolates were examined for their ability to autoaggregate according to the previous protocol [29]. Bacteria were grown overnight in THB medium, washed, and resuspended in sterile distilled water to an OD_600nm_ of 0.3. The degree of autoaggregation of all isolates was determined using the equation: % autoaggregation = (((OD_600nm_ at T_0_-OD_600nm_ at T_60min_)/ OD_600nm_ at T_0_) × 100). OD_600nm_ was recorded following a low-speed centrifugation at 400 *g* for 2 min. Assays were run in triplicate and the means ± SD of three independent experiments were calculated.

#### Far red labeling of *S. suis*

Stocks of *S. suis*, labeled with CellTrace Far Red fluorescent dye (Thermo Fisher Scientific, C34564) (S. suis*FR) as our previous protocol [17], were generated using exponential phase THB cultures (OD_600_ = 0.5). Bacteria were harvested from 8 mL of these cultures (2500 × *g*, 10 min, 4 °C) and washed twice with PBS before resuspending the pellet in 1 mL PBS and adding 1 µL of FR stock solution (1 mM in DMSO). After an incubation for 20 min at 37 °C under rotation in the dark, bacteria were washed again with PBS and finally resuspended in 1 mL THB containing 15% glycerol. Aliquots were frozen in liquid nitrogen. Unlabeled stocks were treated the same way without addition of FR.

### Flow cytometry analysis of *S. suis* association with porcine monocytes

Peripheral blood mononuclear cells (PBMCs) were isolated from whole blood by a density gradient separation as described previously [30]. PBMCs (10^7^ cells/mL) were infected with S. suis*FR at an MOI of 1 for 30 min at 37 ℃, whereby *S. suis* had been pre-incubated in porcine serum of colostrum-deprived piglets (CDS). Monocytes were stained using the myeloid marker CD172a-FITCs (BD Pharmingen^™^, 561498, 0.5 mg/mL). Samples were measured by flow cytometry (BD FACS Calibur) and analyzed with FlowJo^TM^_V10 software.

### C3 deposition on the surface of *S. suis*

Deposition of complement on the streptococcal surface was assessed using flow cytometry assays as described previously [23, 31]. Briefly, C3 deposition was investigated by incubating 2 × 10^6^ CFU of *S. suis* in 50 μL of CDS for 30 min at 37 ℃ under rotation (8 rpm). As negative control CDS was incubated for 30 min at 56 ℃ to inactivate all complement factors. Staining of C3-labeled bacteria was conducted with 200 µL of a 1:150 diluted FITC-labeled cross-reactive rabbit anti-human C3c antibody (Dako, F020102-2, 3 *g*/L) for 1 h at 4 ℃. Samples were measured using BD FACS Fortessa and analyzed using FlowJo^TM^_V10 software. Results of complement binding assay are presented a fluorescence index (FI; percentage of positive bacteria multiplied by the geometric mean fluorescence intensity) in arbitrary units [32, 33].

### Bactericidal assays in whole blood

Comparative analysis of survival of *S. suis* strains was conducted in heparinized porcine blood samples drawn from 8-week-old piglets originating from a conventional farm. Collection of blood was approved by the state Saxony, Germany, under the permit number A09/19. Briefly, 500 µL of heparinized blood were infected with 5 × 10^5^ CFU using stocks of frozen bacteria with 15% glycerol after thawing. The specific bacterial contents (CFU/mL) were determined through plating of serial dilutions after 0 min and 120 min of incubation at 37 ℃. The survival factor (SF) represents the ratio of the CFUs after 120 min to the CFUs at time zero [6].

C3 deposition on the surface of bacteria was conducted parallelly by using the plasma samples of the blood used also in the bactericidal assays*.* The plasma samples were collected prior to incubation with *S. suis* strains, frozen in liquid nitrogen and stored in −80 ℃.

### Cytokine quantification

DuoSet ELISA kits for porcine tumor necrosis factor (TNF)-α and interleukin (IL)-1β were purchased from R&D Systems (DY690B and DY681) and performed essentially according to manufacturer’s recommendations. The analysis was conducted with plasma obtained before and after infection with *S. suis* in bactericidal assays as described above. The streptavidin–horseradish peroxidase used to couple the detection antibodies, was detected with a 3,3ʹ,5,5ʹ-Tetramethylbenzidin (TMB) solution (SeraCare, Milford, MA, USA, formerly KPL) and the reaction was stopped after 20 min with 1 M H_3_PO_4_ (Roth, 6366.1). OD values were measured with a microplate reader SpectraMax 340PC384 (Molecular Devices, LLC San Jose, CA, USA) at 450 and 630 nm as a background reference and analyzed with SoftMax^®^ Pro v5.0 software (Molecular Devices, LLC) [17, 30].

### Oxidative burst experiment

Measurement of oxidative burst and the association of *S. suis* with porcine granulocytes was essentially conducted as described before [34]. Briefly, a total of 5 × 10^5^ CFU of the indicated S. suis*FR stocks were added to 150 µL whole blood of 8-week-old piglets. Positive controls were incubated with 1 μg/mL PMA (product no. 79346-1MG; Sigma-Aldrich). After 20 min of incubation at 37 ℃ dihydrorhodamine123 (DHR123, Sigma, D1054) was added to stain reactive oxygen species (ROS) within the granulocytes. While reacting with ROS, DHR123 is oxidized to fluorescent rhodamine123 (Rho123). Samples were measured by flow cytometry (BD FACSCalibur) and analyzed with FlowJo^TM^_V10 software.

Bactericidal assays were always conducted in parallel with oxidative burst experiments, meaning that 300 µL samples containing fresh heparinized blood and *S. suis* strain (1 × 10^6^ CFU) were divided in two immediately after the addition of bacteria. Whereas one half of the sample was used for oxidative burst experiments, the other half was used for bactericidal assays [18].

### Statistical analysis

Statistical analysis was performed using Prism software, version 8 (GraphPad, San Diego, CA, USA). Normality was tested by Shapiro–Wilk test. Differences between multiple groups were determined using ANOVA followed by Tukey’s multiple comparisons test or Friedman test followed by Dunn’s multiple comparisons test, respectively. A confidence interval of 95% was chosen for all analysis. All figures and data in the text are represented as the means and standard deviations (SD). Probabilities were considered as indicated in the figure legends and supplementary materials.

## Results

### Surface hydrophobicity and autoaggregation of different *S. suis* strains

**Figure 1:**
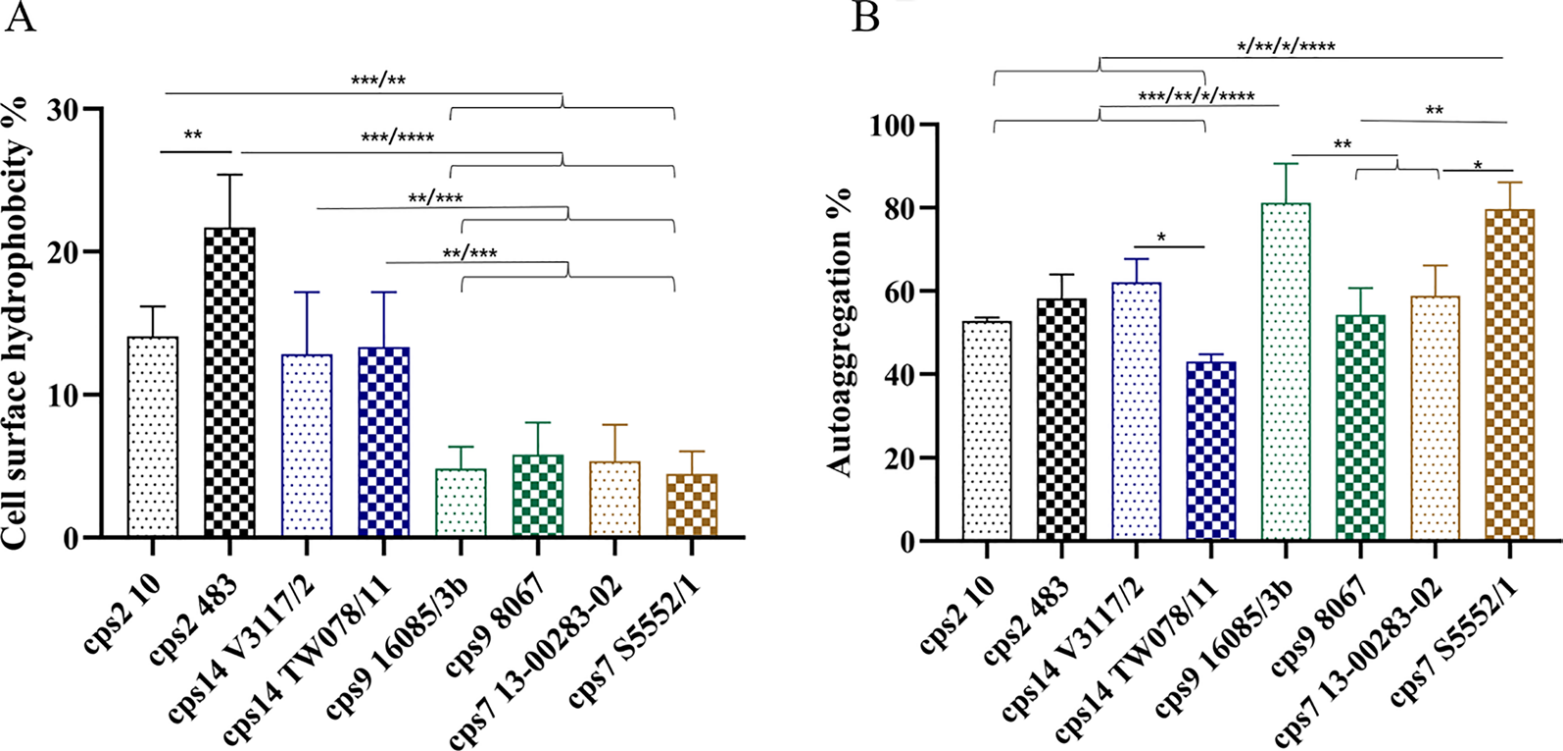
**Cell surface hydrophobicity (A) and autoaggregation (B) of** ***S. suis* strains belonging to different serotypes.** **A** Hydrophobicities were examined by MATH assay. Bars indicate the standard deviation from six independent experiments. **B** Quantitative autoaggregation assay. Bars represent standard deviations from three independent experiments. For statistical analysis, one-way analyses of variance (ANOVAs) with Tukey’s multiple comparisons tests were performed. Differences that are not indicated are not significant. Significant differences are indicated (*p < 0.05, **p < 0.01, and ***p < 0.001). Brackets (}) refer to differences to each column below the bracket.

The composition and thickness of the capsule is likely a major factor determining the physical properties of the surface of *S. suis*. However, expression of surface-associated proteins as well as teichoic acids might also play a substantial role. We compared cell surface hydrophobicity and autoaggregation of 8 *S. suis* strains to characterize them in more detail in this study. As shown in Figure 1A, *cps*2 and 14 strains exhibited significantly higher hydrophobicity than the *cps*9 and 7 strains. No differences in hydrophobicity were recorded between the two strains of *cps*14, 9, and 7. However, the *cps*2 strain 483 belonging to ST28 had a significantly higher surface hydrophobicity than the ST1 strain 10 of *cps*2, which showed values comparable to the *cps*14 strains. Autoaggregation showed a different pattern than cell surface hydrophobicity as significant differences between the two strains within *cps*14, 9, and 7 were recorded (Figure 1B). The *cps*9 strain 16085/3b showed with a mean of 81%, SD (9.35%) the highest level of autoaggregation.

### *S. suis* strains exhibit differences in the association with leukocytes

**Figure 2:**
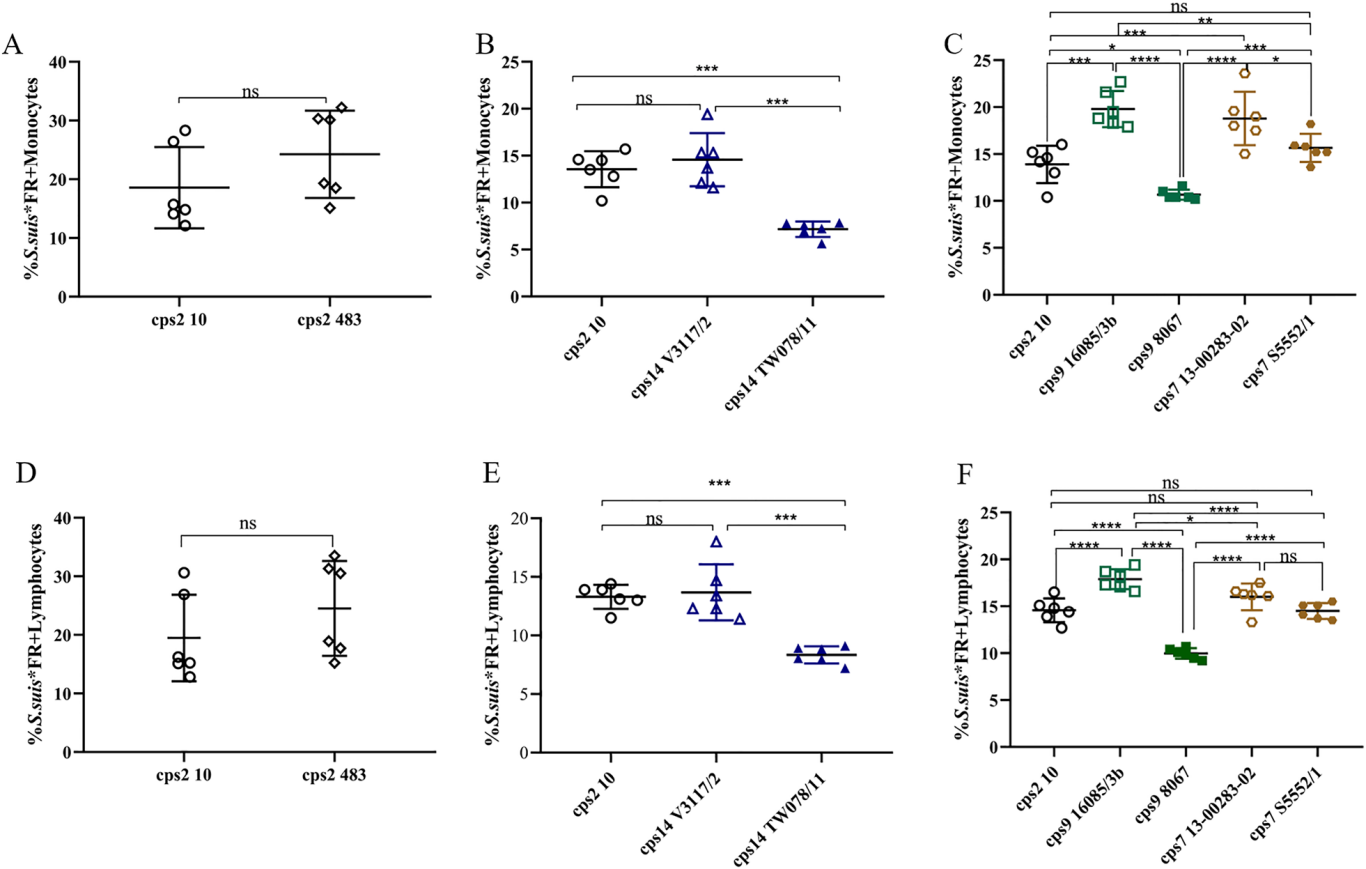
***S. suis* strains show differences in the level of association with monocytes and lymphocytes.** Porcine peripheral blood mononuclear cells (PBMCs) freshly isolated from porcine blood were incubated with Far Red-labeled *S. suis* strains (S. suis*FR) at an MOI of 1 for 30 min at 37 ℃, whereby S. suis*FR had been pre-incubated in serum of colostrum-deprived piglets (CDS) for 30 min (*cps*2, *cps*14, *cps*9, and *cps*7). Monocytes were stained using the myeloid marker CD172a-FITCs and samples were measured by flow cytometry. The levels of associations of *S. suis* strains with monocytes (**A**–**C**) and lymphocytes (**D**–**F**) are shown. Each symbol refers to a different piglet. Data shown in one graph was obtained in one experiment. Horizontal lines and error bars represent mean values and SDs. For statistical analysis, paired t-test (*cps*2 strains 10 vs 483) or one-way analyses of variance (ANOVAs) with Tukey’s multiple comparisons test (*cps*2 strain 10 vs *cps*14 strains or *cps*2 strains vs *cps*9 vs *cps*7 strains) were performed. Significant differences are indicated (*p < 0.05, **p < 0.01, ***p < 0.001, and ****p < 0.0001).

Based on the modified Trojan horse theory, *S. suis* binds to the surface of monocytes during bacteremia and uses these monocytes to breach the blood–brain or blood-cerebrospinal-fluid barrier [13]. Expression of the capsule and D-alanylation of lipoteichoic acid are known to influence association with monocytes [17]. Furthermore, antibodies and deposition of C3 might influence association with leukocytes. In the experiment shown in Figure 2, we asked if the selected strains show differences in the association with leukocytes independent of the presence of specific antibodies. The latter was assured by using serum drawn from piglets prior to colostrum uptake (CDS). We isolated porcine PBMCs from freshly obtained blood and infected them with S. suis*FR strains at an MOI of 1. The myeloid marker CD172a was used to distinguish monocytes from lymphocytes (Additional file 1) and samples were analyzed for FR positive monocytes or lymphocytes by flow cytometry (Figures 2A–C). As the capsule is likely a key determinant of monocyte association, we hypothesized that two strains of the same serotype should show a comparable phenotype. However, significant differences were recorded between the two strains of *cps*14 and also between the two *cps*9 strains (Figure 2). Of note, 19.8% (SD 1.9%) of *cps*9 strain 16085/3b were associated with monocytes whereas this was only observed for 10.7% of *cps*9 8067 (SD 0.5%) bacteria. Interestingly, *cps*2 strain 10 and *cps*14 strain V3117/2, both belonging to ST1, exhibited comparable levels of monocyte association. These results indicate that other factors but the composition of the capsule play a major role in association with monocytes. In general, the differences in monocyte association between two strains were approximately also found in lymphocyte association except that differences between the two *cps*7 strains were not significant.

### Labeling with complement in the absence of specific antibodies

Deposition of C3 on the bacterial surface limits survival of *S. suis cps*2 in porcine blood through induction of opsonophagocytosis [18]. Loss-of-function experiments have shown that the capsule and different surface-associated proteins of *cps*2 are involved in complement evasion [16, 35, 36]. We compared the *S. suis* strains in a flow cytometric assay designed to detect C3 on the bacterial surface after incubation in serum lacking specific antibodies to negate the effect of differences in the level of antibodies specific for each individual strain that would interfere with complement activation. Therefore, the C3 deposition measured in this assay is not due to the classical pathway but to the alternative or lectin pathway of complement activation. Overall the percentage of *S. suis* bacteria labeled with C3 was below 8% and the FI of C3 binding below 150 in all investigated strains. Of note, the two *cps*9 strains exhibited a comparable level of C3 deposition although association with monocytes was found to be significantly different between the two strains.

### Labeling with complement in the presence of specific antibodies

Diseased piglets in the field generally carry IgG and often also IgM antibodies binding to the surface of *S. suis*. Antibody-mediated activation of the complement system is an important mechanism in the control of *S. suis* bacteremia [18]. We investigated survival and C3 labeling of the different *cps*2*,* 9, and 14 strains in blood and plasma, respectively (*cps*7 was not investigated as it was known that these strains are efficiently killed in blood of pigs at this age [6]). The samples were drawn from 8-week-old piglets from a herd known to be infected with different *cps* such as 1, 1/2, 2, 7 and 9 [37]. Furthermore, piglets at this age in this herd carry IgM antibodies binding to *cps*7, *cps*1 and *cps*14 [6, 25]. The unencapsulated mutant of *cps*2 strain 10 (10cpsΔEF) was nearly completely killed in the blood of all piglets. *cps*2 strain 10, both *cps*14 strains and *cps*9 strain 8067 were also efficiently reduced in number in the blood of all piglets except one as the mean bacterial SF was below 0.4 (Figure 4A). In contrast, *cps*9 16085/9b showed high proliferation rates with a mean SF of 27.5 (SD = 23.4). Accordingly, SF were significantly different between the two *cps*9 strains. Though differences were not significant between the two *cps*2 strains, *cps*2 strain 483 survived in the blood of the majority of piglets (SF > 1) in contrast to *cps*2 strain 10. Complement deposition on the bacterial surface was by far the highest in the unencapsulated mutant 10*cps*ΔEF: The FI of C3 labeling obtained values above 3000, approximately ten times higher in comparison to the encapsulated wt strains. The respective FI of C3 labeling was comparable between the different *cps*2 and *cps*14 strains with values around 300. The lowest values of C3 labeling were found for the *cps*9 strain 16085/3b which was the only wt strain of the investigated strains proliferating efficiently in the blood of all piglets. We conducted a correlation analysis of bacterial survival and complement deposition on the bacterial surface. As shown in Figure 4C, the FI of C3 labeling showed a strong negative correlation with the SF as the Spearman correlation coefficient obtained a value of −0.72 (Figure 4C). The FI of C3 labeling of bacteria in plasma was below 250 if the respective *S. suis* strain showed a SF above 1 in the respective blood sample (Figure 4C). However, a number of blood samples induced killing of a *S. suis* wt strain though FI of C3 binding was below 100 in the respective plasma samples.

### Induction of IL-1β and TNF-α in porcine blood infected with different *S. suis* strains

We measured IL-1β and TNF-α in plasma samples of the bactericidal assays depicted in Figure 4 as lead cytokines of inflammasome activation and inflammation, respectively. Both cytokines were induced in all infected blood samples. Furthermore, we observed a substantial overlap in the values of IL-1β concentration between the different *S. suis* strains (Figure 5A). *cps*2 and *cps*14 strains induced comparable levels of IL-1β, although *cps*2 strain 483 proliferated in most samples and *cps*2 encapsulated strain 10 and its unencapsulated mutant 10*cps*ΔEF were killed (Figure 4A). The TNF-α obtained mean values not higher than 20 ng/mL within 2 h after infection of porcine blood. Of note, the mean concentration of this proinflammatory cytokine was even below 10 ng/mL in the case of the two *cps*9 strains and therefore significantly lower than in the blood samples infected with any of the other strains. There were no significant differences in the induction of IL-1β or TNF-α between strains of the same serotype (Figure 5). It is worth noting that the significant difference of induction of IL-1β or TNF-α were not recorded between *cps*9 two strains although *cps*9 strain 16085/3b proliferated in the blood samples of all six piglets and strain 8067 was efficiently killed (Figure 4A). Spearman correlation showed that there were no correlations between the induction of IL-1β or TNF-α with SFs of *S. suis* strains (Additional file 2).

### Induction of ROS in porcine blood infected with different *S. suis* strains

To investigate the role of induction of the oxidative burst in killing of *S. suis*, bactericidal and oxidative burst assays with FR-labeled *S. suis* strains were conducted parallelly by using the same blood. The blood samples were drawn from 9-week-old piglets. In all blood samples *S. suis* infection induced detectable oxidative burst rates (Rho123^+^ granulocytes). The unencapsulated mutant 10*cps*ΔEF induced the highest frequencies of Rho123^+^ granulocytes with a mean of 7.2% (SD = 4.6%), *cps*14 strain V3117/2 and *cps*9 strain 16085/3b also induced strong ROS production (> 5%), *cps*7 strain S5552/1 showed the lowest oxidative burst rates (mean = 1.1%, SD = 0.56%), and the other *S. suis* wt strains showed moderate levels of ROS induction from 1.9 to 4.3% (Figure 6A). At the same time, we observed the unencapsulated mutant strain 10*cps*ΔEF, *cps*14 strains V3117/2 and TW078/11, *cps*9 strain 8067 and *cps*7 strain 13-00283-02 were effectively killed by the all six blood samples as the mean bacterial SF below 0.3 (Figure 6B). In contrast, *cps*2 strain 10 survived in the blood of piglets with the mean of SF > 1.0. Of note, *cps*9 strain 16085/3b proliferated only in one sample and was killed in the other 5 blood samples (Figure 6B). To answer the question, whether higher oxidative burst level result in an increased killing of the bacteria in whole blood, we conducted a correlation analysis of bacterial survival and *S. suis* induced oxidative burst rates. As shown in Figure 6C, the percentage of *S. suis*-FR*^+^Rho123^+^ granulocytes negatively correlated with the bacterial survival factors (Spearman r = −0.51with *p* < 0.0001; Figure 6C). In the case that ROS induction was above 4%, the respective encapsulated *S. suis wt* strain was killed with a SF below 0.4 in the respective blood sample (Figure 6C). However, a number of blood samples induced killing of *S. suis* wt strains although Rho123 positive granulocytes were below 4% in the respective blood samples.

## Discussion

**Figure 3:**
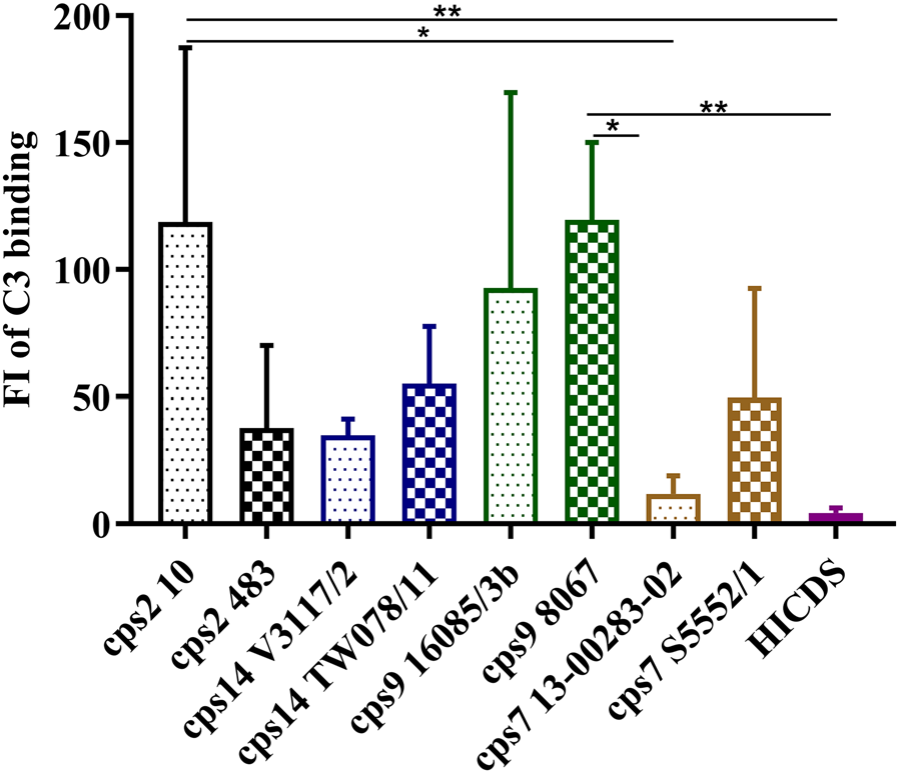
**C3 deposition on the surface of** ***S. suis* strains in serum containing no specific antibodies.** The indicated *S. suis* strains of serotypes 2, 14, 9, and 7 were incubated in serum of colostrum-deprived piglets (CDS) for 30 min, followed by staining of C3 with a cross-reactive FITCs conjugated anti-human C3c antibody and measurement of C3 antigen bound to the bacterial surface by flow cytometry. The results were expressed as the fluorescence index (FI) of bacteria with C3 bound to their surface. HICDS (heat-inactivated CDS) was used as a negative control. Flow cytometry data were analyzed using Dunn’s multiple comparisons test and presented as the mean values with SDs. The experiment was repeated 5 times. Differences that are not indicated are not significant. Significant differences are indicated (*p < 0.05, and **p < 0.01).

**Figure 4:**
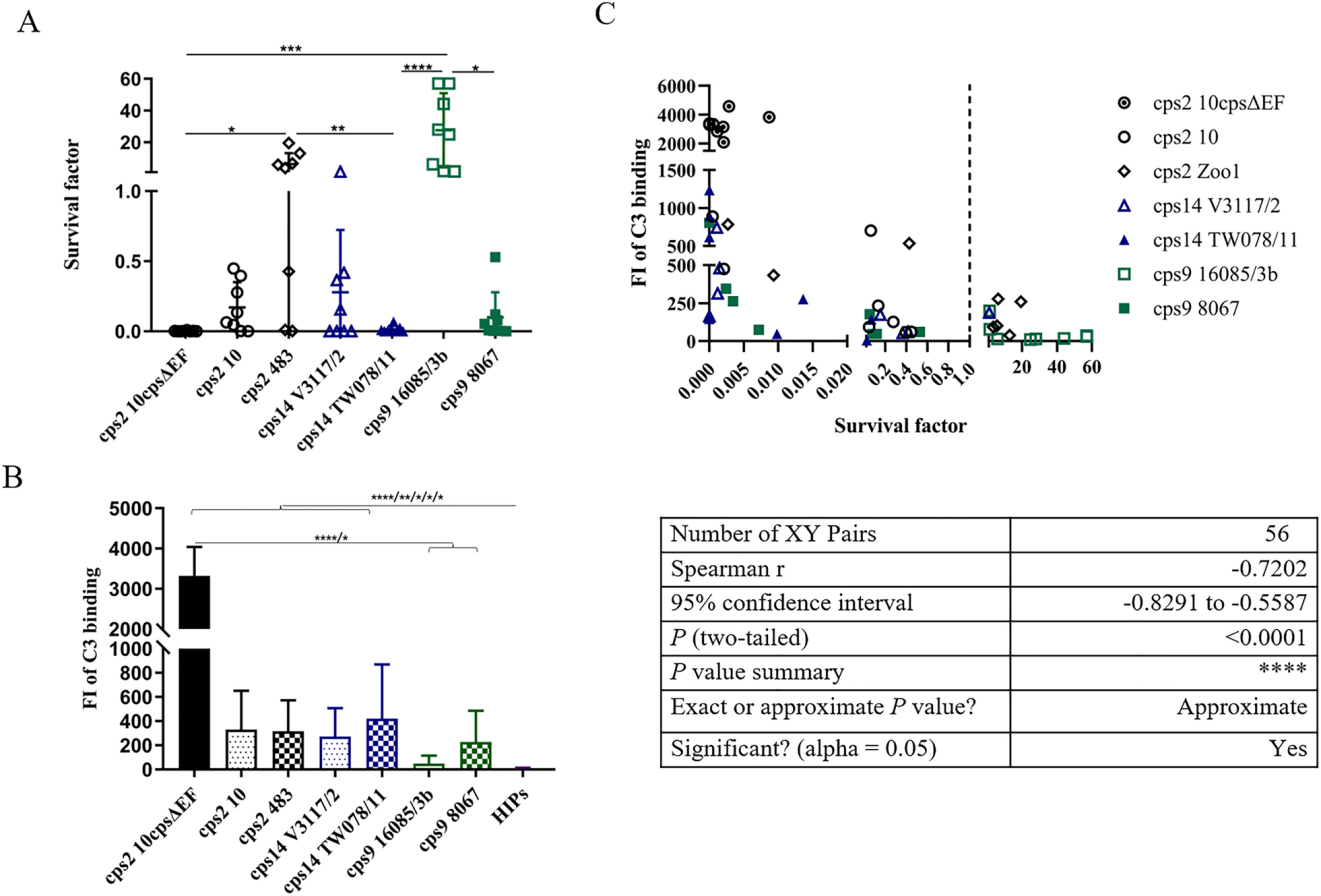
**Killing of** ***S. suis* strains in porcine blood (A) is associated with the deposition of C3 (B) on the bacterial surface.** Survival of the different strains was determined in heparinized blood samples of 8-week-old piglets in vitro (**A**). The specific bacterial contents (CFU/mL) were determined through plating of serial dilutions after 0 min and 120 min of incubation at 37 ℃. The survival factor (SF) represents the ratio of the CFUs after 120 min to the CFUs at time zero. The FI of C3 deposition on the bacterial surface (**B**) of the indicated *S. suis* strains after incubation in plasma samples obtained from the blood used also in the bactericidal assay shown in A. Staining of C3 was conducted with a cross-reactive FITC-conjugated anti-human C3c antibody and measured by flow cytometry. HIPs (heat-inactivated plasma) was used as a negative control. Flow cytometric data and survival data was analyzed using Friedman test followed by Dunn’s multiple comparisons test and is presented as mean values with SDs. Error bars represent SD and are too small to be visible outside the symbol when not present. Significant differences are indicated with the star symbols (*p < 0.05, **p < 0.01, and ***p < 0.001). Spearman correlation was calculated between C3 deposition on the streptococcal surface with the respective SFs of the indicated *S. suis* strains in blood. The correlation between SFs and the FI of the samples (**C**) is shown.

*Streptococcus suis* is one of the most important porcine pathogens and an emerging human pathogen. To cause invasive infections, *S. suis* needs to enter the bloodstream, evade the host immune system, proliferate and disseminate along with the bloodstream [2, 12]. For *S. suis cps*2 of CC1, it has been shown that the complement system is an important host defense mechanism limiting bacterial survival in blood and dissemination [14–18]*.* Although it appears likely that this is true for other serotypes and clonal complexes as well, there are few experimental data on this issue for other major pathotypes. The results of this study indicate that antibody-mediated C3 binding restricts also survival of other *S. suis* pathotypes in porcine blood, because the FI of C3 deposition on the bacterial surface were generally much higher after incubation in plasma of conventional piglets in comparison to incubation in serum collected prior to colostrum uptake (compare Figures 3 and 4) and the FI of C3 deposition on the streptococcal surface showed a strong negative correlation with the bacterial survival factor in porcine blood in the analysis of the entire data for *cps*2, 14, and 9.

The interactions between *S. suis* and different immune cell types, including monocytes, neutrophils and lymphocytes in the blood, is considered to affect the progress of infection [2, 12]. Bacterial cell surface properties, such as hydrophobicity and autoaggregation, play important roles in bacteria-host cell encounters [38, 39]. Comparing encapsulated with unencapsulated strains, previous studies have demonstrated that encapsulation of *S. suis* is associated with lower surface hydrophobicity [17, 29, 40]. As shown in Figure 1A, *cps*2 and *cps*14 strains showed higher hydrophobicity levels (above 12%) than strains of *cps*9 and *cps*7 (below 6%). This is in part in agreement with results of Okura et al. [40], suggesting that a switch of *cps*2 to *cps*9 is associated with a decrease in surface hydrophobicity. Another study [41] reported a low cell surface hydrophobicity (≤ 11%) for *cps*2 strains of sequence types 1 and 28, which is lower than the values determined for the *cps*2 strain of sequence type 28 in our study.

**Figure 5:**
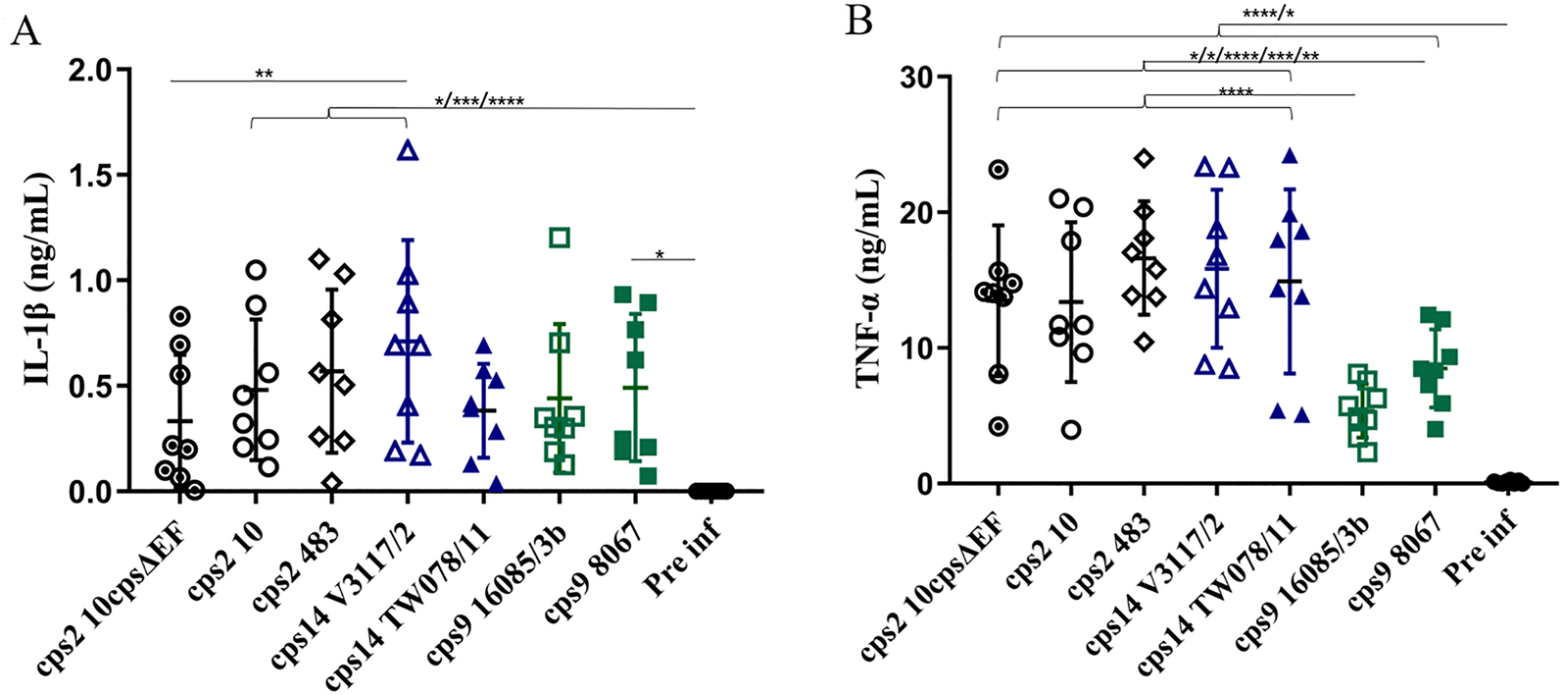
**Concentrations of IL-1β (A) and TNF-α (B) in in vitro infected porcine blood samples.** The concentrations of these proinflammatory cytokines were measured by ELISA 2 h after in vitro infection of blood samples drawn from 8-week-old piglets (*n* = 8) with the indicated *S. suis* strains of serotypes 2, 14, and 9. The control plasma samples were collected prior to in vitro infection with *S. suis* strains (pre inf). Horizontal lines and error bars represent mean values and SDs, respectively. The limit of detection was 0.008 ng/mL for TNF-α and IL-1β and all pre-inf samples lay below that limit. The concentrations were analyzed by using Friedman followed by Dunn’s multiple comparisons test and ANOVAs with Turkey’s multiple comparisons tests, respectively. Differences that are not indicated are not significant. Significant differences are indicated (*p < 0.05, **p < 0.01, ***p < 0.001, and ****p < 0.0001). Brackets (}) refer to differences to each column below the bracket.

In the autoaggregation assay, all 8 strains demonstrated autoaggregation. Strains of *cps*9 and *cps*7 showed slightly higher levels than the two *cps*2 strains (Figure 1B). A previous study showed that non-typeable *S. suis* isolates were able to autoaggregate to various extents, while *cps*2 strains could not [29]. The authors showed that autoaggregation, hydrophobicity, and adherence to host cells were all increased in unencapsulated strains [29]. Our data suggests that among encapsulated *S. suis* strains these properties might not show comparable patterns (Figures 1 and 2) and differences cannot be simply explained by different capsule types. Besides the capsule, adhesion factors like antigen I/II can be involved in autoaggregation of *S. suis*, as it was shown for a *cps*9 strain [42]. The levels of association with monocytes were comparable between the two investigated *cps*2 strains 10 and 483, although cell surface hydrophobicity was significantly different (Figure 1A). On the other hand, the *cps*9 and *cps*7 strains showed significant differences in the percentage of monocytes associated with the respective strain despite comparable hydrophobicity of their surface. This indicates that the surface hydrophobicity does not play a crucial role in determination of the association to monocytes. Interestingly, considering the *cps*2, 14, and 9 strains the autoaggregation behavior follows a pattern comparable to the results obtained for monocyte association, with higher autoaggregation levels and higher numbers of monocytes associated with strains V3117-2 (*cps*14) and 16085/3b (*cps*9) compared to the second *cps*14 and *cps*9 strain, respectively. In addition to these nonspecific physicochemical properties of bacterial cell surface, specific interactions between the streptococci and host cells can significantly influence the outcome of their contact [2, 12]. Macrophages, monocytes, and polymorphonuclear leukocytes have been shown to express the complement receptor 1 which binds to complement protein C3b on the bacterial surface. We asked if enhanced C3 deposition on the streptococcal surface is associated with increased binding to leukocytes. Our data shows that only very little C3 is detectable on the surface of the different encapsulated *S. suis* strains in the absence of specific antibodies (Figure 3). Although *cps*9 strain 16085/3b exhibited significantly higher levels of association with monocytes and lymphocytes than *cps*9 strain 8067, there was no significant difference in C3 binding between the two *cps*9 strains in the absence of specific antibodies (Figure 3). In addition to the capsule, surface-associated or secreted proteins might also contribute to the attachment to monocytes and explain the difference between the two *cps*9 strains [15, 43]. Though *cps*9 strain 16085/3b showed in comparison to the other strains significantly increased association with monocytes and proliferation in the investigated blood samples, respective TNF-α plasma concentrations were rather low (Figure 5B). We speculate that interaction of strain 16085/3b with monocytes is associated with modulation of monocyte functions such as secretion of TNF-α. Interestingly, major differences in modulation of monocyte function were demonstrated for different isolates of *Staphylococcus aureus* [44]. Meijerink et al. [45] investigated the interaction of different *S. suis* serotypes with human monocyte-derived dendritic cells and found the *cps*2 strain to induce lower amounts of pro-inflammatory cytokines and cause less activation of the cells than the strains of *cps*1, 4, 7, 9 and 14. The *cps*2 strain 10 and *cps*9 strain 8067 were also used in our study. In contrast to Meijerink et al. [45], we found significantly higher levels of TNF-α induced by strain 10 compared to strain 8067 in porcine blood. Overall, our values were higher, which can be explained by the likely presence of antibodies in porcine blood that opsonize *S. suis* and stimulate cytokine production of monocytes.

Once *S. suis* enters the bloodstream during the process of infection, the complement system participates in first line of defence in blood and promotes the rapid elimination of bacteria. Evasion of complement-mediated immunity is important for *S. suis* infection, and differences in susceptibility to complement correlates with the virulence of different *S. suis cps*2 strains as described in previous investigations [14, 35, 46]. Our data suggest that complement-mediated opsonophagocytosis is not only important for control of *cps*2 infection, since high deposition of C3 on the bacterial surface was negatively correlated with bacterial survival across the whole spectrum of samples originating from serotypes 2, 14, and 9. For example, *cps*9 strain 16085/3b showed very little complement deposition and high proliferation under the chosen experimental infection, while *cps*9 strain 8067 was killed and obtained higher values for C3 binding. Nevertheless, our present data also showed killing of *S. suis* strain 10 in blood without high FI of C3 binding (Figure 4C), indicating that complement-independent killing mechanism exist. This is in accordance with our previous study, where complement was not crucial for the killing of *S. suis* strain 10 in blood reconstituted with hyperimmune serum raised against *cps*2 strain 10 [18]. However, we could generally observe killing of *S. suis* wt strains, when complement was prominently activated.

The induction of inflammatory cytokine responses upon infection of both human and porcine whole or diluted blood with *S. suis* strains has been demonstrated in previous studies [47, 48]. Our investigation showed that *S. suis* strains of *cps*2, 14, and 9 induced IL-1β and TNF-α following in vitro infection of porcine blood as previously reported in human whole blood [47]. Hohnstein et al. found that the induction of pro-inflammatory cytokines by encapsulated and unencapsulated *S. suis cps*2 in vitro in whole blood is similar, even though they observed differences in bacterial survival [30]. In our present study, IL-1β and TNF-α induction were not associated with killing of *S. suis*. Accordingly, Hohnstein et al. found that TNF-α does not contribute to bacterial killing in whole blood in vitro [30].

**Figure 6:**
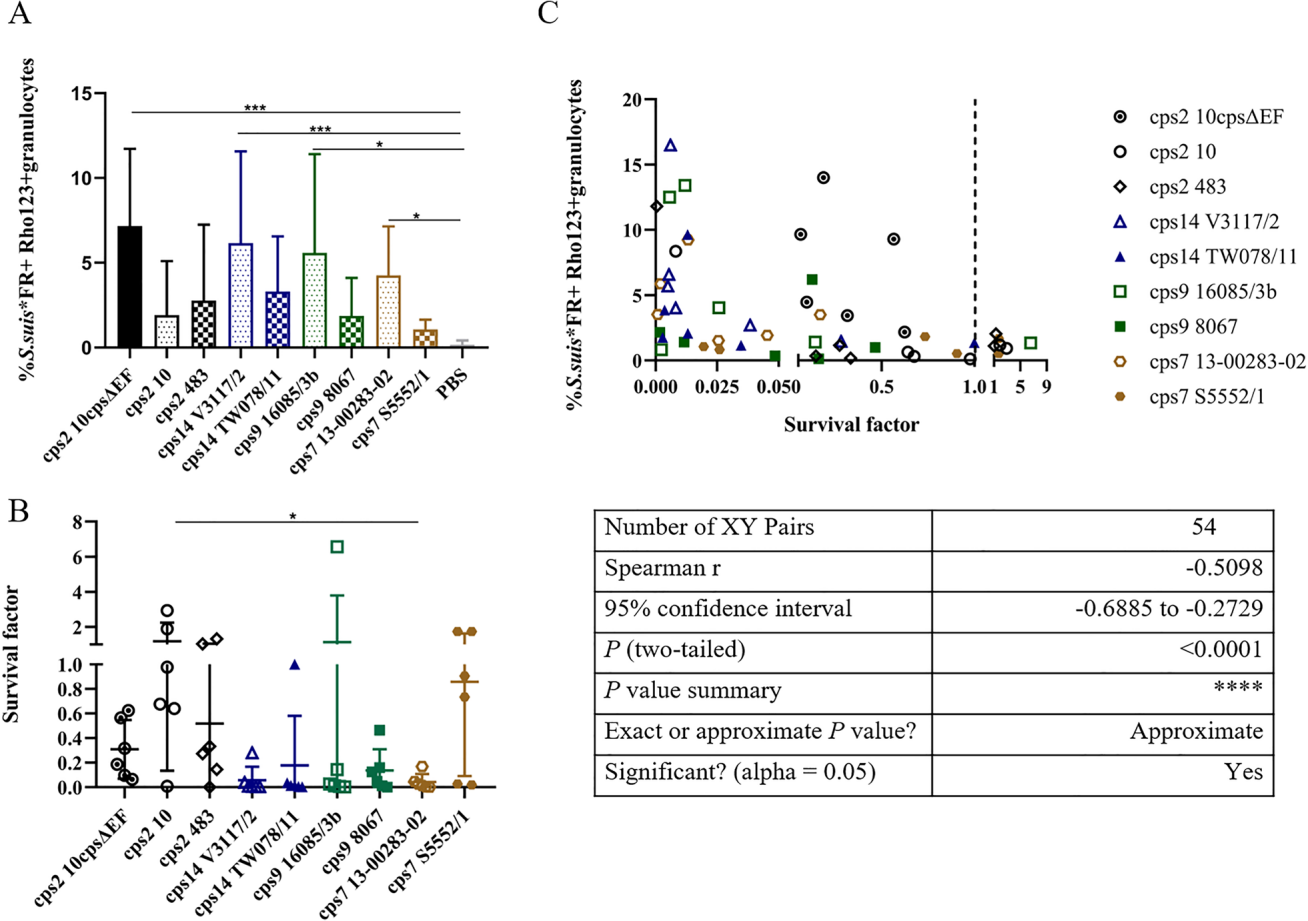
**Oxidative burst of granulocytes (A) and survival factors (B) of *S. suis* strains in porcine blood.** Bactericidal assays were conducted in parallel with oxidative burst experiments in blood of 9-week-old piglets (*n* = 6). After adding 1 × 10^6^ CFU of *S. suis* to 300 µL of heparinized blood, the samples were divided into two immediately. For determination of oxidative burst, one sample of each pair was incubated for 20 min at 37 ℃ and for a further 10 min after the addition of DHR 123 (5 μg/mL). As DHR 123 reacts to Rho 123 in the presence of reactive oxygen species, the oxidative burst is shown as a percentage of Rho123 positive granulocytes. Following erythrocyte lysis, the samples were directly measured by flow cytometry (**A**). SFs (**B**) were determined in the other sample of each pair after 2 h of infection (150 μL of heparinized blood infected with 5 × 10.^5^ CFU of the indicated *S. suis* strain). For statistical analysis, Friedman followed by Dunn’s multiple comparisons test was performed both in oxidative burst experiment and bactericidal assays among 9 *S. suis* strains including 8 wt strains and 1 unencapsulated isogenic mutant 10cpsΔEF. Differences that are not indicated are not significant. Significant differences are indicated (*p < 0.05, **p < 0.01, and ***p < 0.001). Spearman correlations between *S. suis* induced oxidative burst rates with the respective streptococcal SF** (C)**.

Our previous investigation showed that induction of the oxidative burst in blood granulocytes also plays an important role in the killing of *S. suis* [18]. In the present study, ROS induction in blood granulocytes was detectable after infection of porcine blood with different *S. suis* strains in vitro. Infected porcine blood samples with high levels of ROS induction (> 5%) showed also prominent bactericidal activity against *S. suis cps*2, the unencapsulated mutant 10*cps*ΔEF and *cps*14 strainV3117/2 (Figure 6). This is in line with our previous results indicating association of levels of *S. suis* induced ROS in granulocytes and bactericidal activity of porcine blood [34].

There is an urgent need to expand *S. suis* pathogenesis research to other *cps* but 2 as this pathogen is very diverse and other *cps* contribute substantially to major disease problems in the pig industry worldwide. Accordingly, we compared strains belonging to different *cps* and sequence types in this study. However, the results obtained for a single strain with a distinct combination of these characteristics cannot necessarily be applied to all strains belonging to the same *cps* and sequence type as shown for *cps* 2 strains of sequence type 28 [49]. Our results suggest on the one hand that antibody-mediated C3 deposition on the bacterial surface and ROS induction in blood granulocytes are generally important for control of *S. suis* bacteremia [15, 18, 34]. On the other hand, our study revealed significant differences in monocyte association between different virulent strains, even within the same *cps*. The latter is in accordance with the concept of different pathotypes of *S. suis* which might use different mechanisms to survive in blood and breach barriers of the host to cause severe pathologies such as meningitis and arthritis.

### Supplementary Information

**Additional file 1: Gating strategy for monocytes (A) and lymphocytes (B) associated with** ***S. suis***. *S. suis* (here strain 10) was labeled with CellTrace Far Red fluorescent dye (*S. suis* FR). PBMCs were freshly isolated from porcine blood. Monocytes were stained using the myeloid marker CD172a-FITCs and samples were measured by flow cytometry (visualization for PBMCs of one animal).

**Additional file 2: Spearman correlation analysis between levels of IL-1β (A) and TNF-α (B) in** ***S. suis* infected porcine blood and survival factors of the indicated strains.** The concentrations of IL-1β and TNF-α were measured by ELISA 2 h after in vitro infection of blood samples drawn from 8-week-old piglets (*n* = 8) with the indicated *S. suis* strains of serotypes 2, 14, and 9 (Figure 5). The specific bacterial contents (CFU/mL) were determined through plating of serial dilutions after 0 min and 120 min of incubation at 37 ℃. The survival factor represents the ratio of the CFUs after 120 min to the CFUs at time zero.

## Data Availability

The raw data supporting the conclusions of this article will be made available by the corresponding author on reasonable request.
