# Correction: Comparative analysis of the interactions of different *Streptococcus suis* strains with monocytes, granulocytes and the complement system in porcine blood

**DOI:** 10.1186/s13567-024-01288-9

**Published:** 2024-03-16

**Authors:** Haodan Zhu, Uwe Müller, Christoph Georg Baums, Sophie Öhlmann

**Affiliations:** 1 Institute of Bacteriology and Mycology, Centre for Infectious Diseases, Faculty of Veterinary Medicine, University of Leipzig, Leipzig, Germany; 2 Institute of Veterinary Medicine, Jiangsu Academy of Agricultural Sciences, Nanjing, China; 3 Institute of Immunology, Centre for Infectious Diseases, Faculty of Veterinary Medicine, University of Leipzig, Leipzig, Germany

**Correction: Veterinary Research (2024) 55:14** 10.1186/s13567-024-01268-z

Following publication of the original article [1], the authors informed us that in the legend of Figure 4C cps2 Zoo1 is identical to cps2 483. Comment: Unfortunately, strain 483 is currently already published in the MLST database with the name Zoo1, though 483 is the name provided in the original publication.
