# Supplementary figures and images for: Comparative analysis of the interactions of different Streptococcus suis strains with monocytes, granulocytes and the complement system in porcine blood
Source: Vet Res. 2024 Feb 5;55:14. doi: 10.1186/s13567-024-01268-z (PMC10845567; doi:10.1186/s13567-024-01268-z)

**Additional file 1. Gating strategy for monocytes (A) and lymphocytes (B) associated with *S. suis*.**

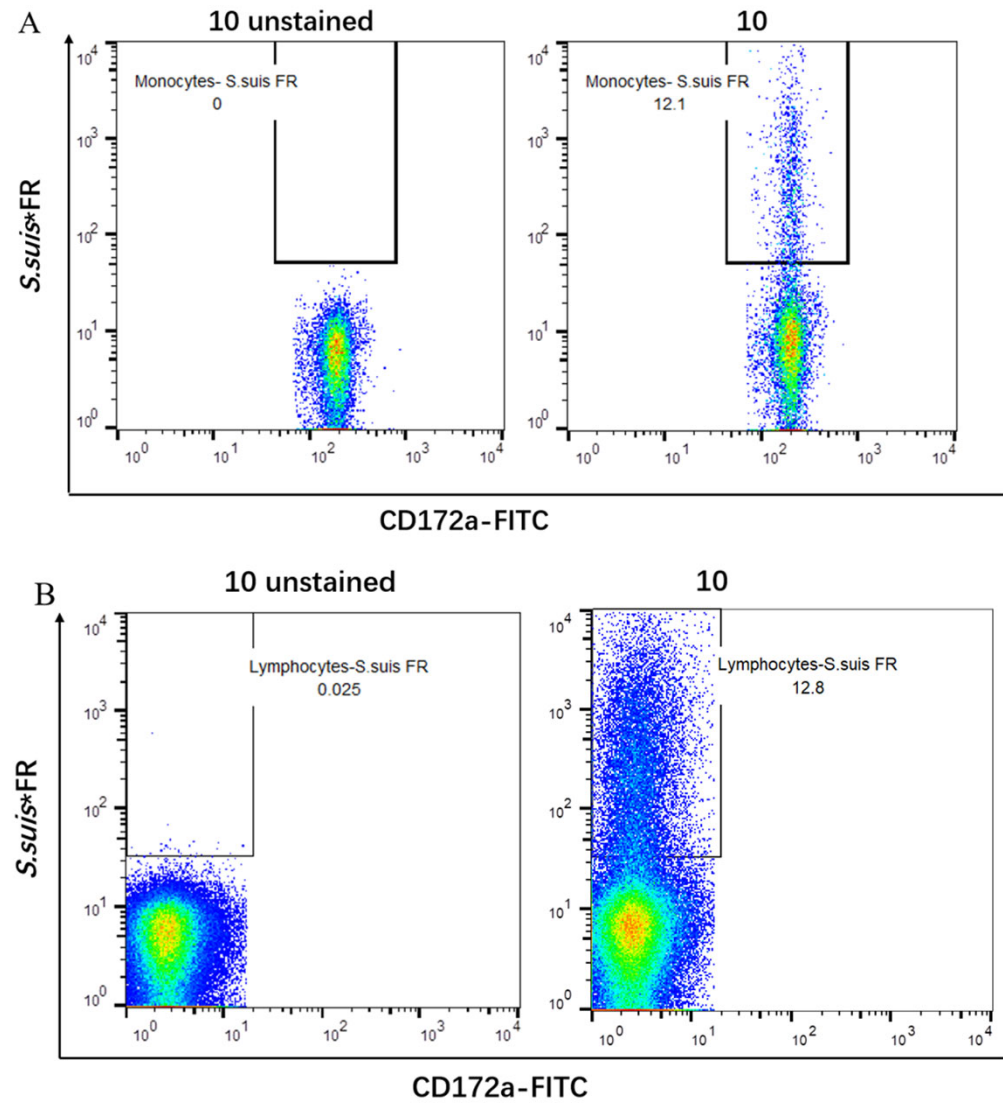

Supplement: Supplementary file 1 — Additional file 1: Gating strategy for monocytes (A) and lymphocytes (B) associated with S. suis. S. suis (here strain 10) was labeled with CellTrace Far Red fluorescent dye (S. suis FR). PBMCs were freshly isolated from porcine blood. Monocytes were stained using the myeloid marker CD172a-FITCs and samples were measured by flow cytometry (visualization for PBMCs of one animal). [file 13567_2024_1268_MOESM1_ESM.pdf]
