# Supplementary material for: Comparative analysis of the interactions of different Streptococcus suis strains with monocytes, granulocytes and the complement system in porcine blood
Source: Vet Res. 2024 Feb 5;55:14. doi: 10.1186/s13567-024-01268-z (PMC10845567; doi:10.1186/s13567-024-01268-z)
Supplement: Supplementary file 2 — Additional file 2: Spearman correlation analysis between levels of IL-1β (A) and TNF-α (B) in S. suis infected porcine blood and survival factors of the indicated strains. The concentrations of IL-1β and TNF-α were measured by ELISA 2 h after in vitro infection of blood samples drawn from 8-week-old piglets (n = 8) with the indicated S. suis strains of serotypes 2, 14, and 9 (Figure 5). The specific bacterial contents (CFU/mL) were determined through plating of serial dilutions after 0 min and 120 min of incubation at 37 ℃. The survival factor represents the ratio of the CFUs after 120 min to the CFUs at time zero. [file 13567_2024_1268_MOESM2_ESM.pdf]

Additional file 2. Spearman correlation analysis between levels of IL-1β (A) and TNF-α (B) in *S. suis* infected porcine blood and survival factors of the indicated strains.

A

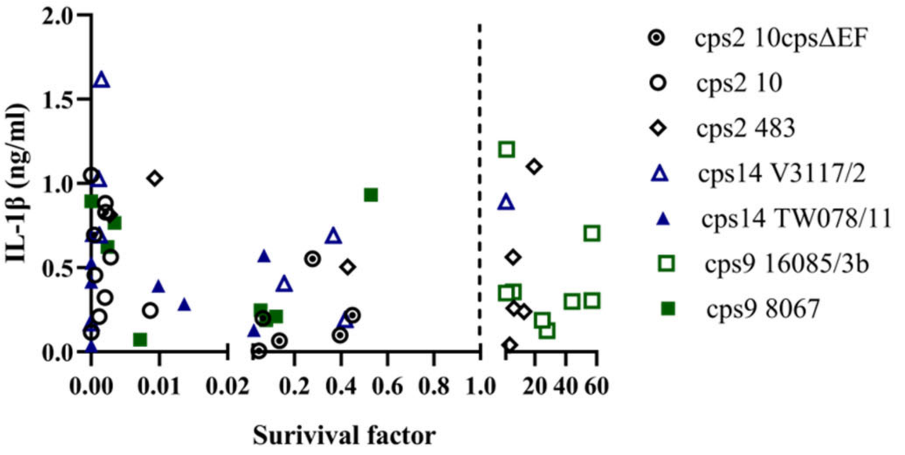

Number of XY Pairs	56
Spearman r	-0.1505
95% confidence interval	-0.4044 to 0.1249
P (two-tailed)	0.2682
P value summary	ns
Exact or approximate P value?	Approximate
Significant? (alpha = 0.05)	No

B

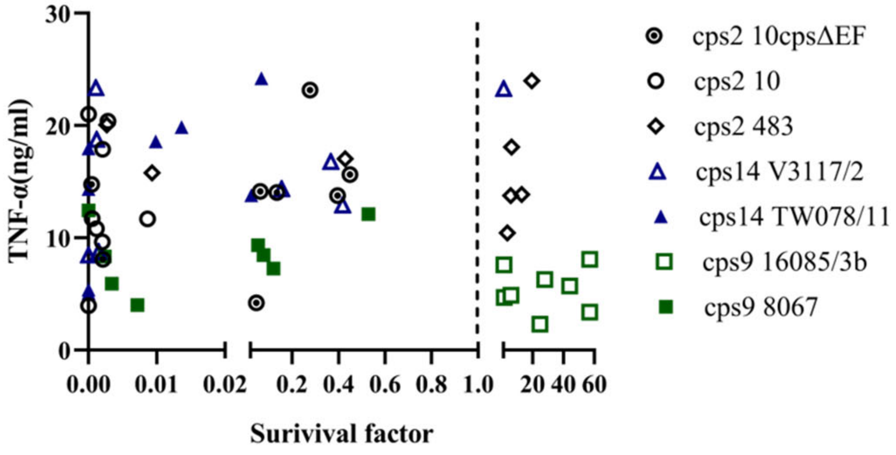

Number of XY Pairs	56
Spearman r	-0.1106
95% confidence interval	-0.3698 to 0.1646
P (two-tailed)	0.4172
P value summary	ns
Exact or approximate P value?	Approximate
Significant? (alpha = 0.05)	No
